# Development and Validation of a Scoring Rubric for Editorial Evaluation of Peer-review Quality: A Pilot Study

**DOI:** 10.5811/westjem.18432

**Published:** 2024-02-28

**Authors:** Jeffrey N. Love, Anne M. Messman, Jonathan S. Ilgen, Chris Merritt, Wendy C. Coates, Douglas S. Ander, David P. Way

**Affiliations:** * Georgetown University School of Medicine, Department of Emergency Medicine, Washington, District of Columbia; † Wayne State University, Department of Emergency Medicine, Detroit, Michigan; ‡ University of Washington, Department of Emergency Medicine, Seattle, Washington; § Alpert Medical School of Brown University, Department of Emergency Medicine, Providence, Rhode Island; ∥ Alpert Medical School of Brown University, Department of Pediatrics & Emergency Medicine, Providence, Rhode Island; ¶ David Geffen School of Medicine at UCLA, Department of Emergency Medicine, Los Angeles, California; # Emory University, Department of Emergency Medicine, Atlanta, Georgia; ** Ohio State University College of Medicine, Department of Emergency Medicine, Columbus, Ohio

## Abstract

**Introduction:**

Despite the importance of peer review to publications, there is no generally accepted approach for editorial evaluation of a peer review’s value to a journal editor’s decision-making. The graduate medical education editors of the *Western Journal of Emergency Medicine* Special Issue in Educational Research & Practice (Special Issue) developed and studied the holistic editor’s scoring rubric (HESR) with the objective of assessing the quality of a review and an emphasis on the degree to which it informs a holistic appreciation for the submission under consideration.

**Methods:**

Using peer-review guidelines from several journals, the Special Issue’s editors formulated the rubric as descriptions of peer reviews of varying degree of quality from the ideal to the unacceptable. Once a review was assessed by each editor using the rubric, the score was submitted to a third party for blinding purposes. We compared the performance of the new rubric to a previously used semantic differential scale instrument. Kane’s validity framework guided the evaluation of the new scoring rubric around three basic assumptions: improved distribution of scores; relative consistency rather than absolute inter-rater reliability across editors; and statistical evidence that editors valued peer reviews that contributed most to their decision-making.

**Results:**

Ninety peer reviews were the subject of this study, all were assessed by two editors. Compared to the highly skewed distribution of the prior rating scale, the distribution of the new scoring rubric was bell shaped and demonstrated full use of the rubric scale. Absolute agreement between editors was low to moderate, while relative consistency between editor’s rubric ratings was high. Finally, we showed that recommendations of higher rated peer reviews were more likely to concur with the editor’s formal decision.

**Conclusion:**

Early evidence regarding the HESR supports the use of this instrument in determining the quality of peer reviews as well as its relative importance in informing editorial decision-making.

## BACKGROUND

Peer review plays a critical role in the traditional paradigm of published scholarship. While peer review is the standard for assessing scholarly submission for publication, the most appropriate means by which to assess the quality of peer review remains unclear.^1^ ^–^ ^8^ This issue is problematic for all the stakeholders of published scholarship. The development of a rigorous and valid tool for editors to assess the quality of peer reviews could help to enhance the peer-review process. This would improve editors’ abilities to stratify the contributions of their reviewer pool, identifying reviewers who deserve outstanding recognition as well as those who could benefit from dedicated mentorship, and inform mechanisms to evaluate the downstream impact of interventions to improve the quality of peer reviews.

Efforts to assess peer-review quality have been challenging. Prior studies have been based primarily on the belief that review evaluation is an objective process.^9^ ^–^ ^12^ Consequently, interventions have been aimed at achieving a high degree of absolute reliability in scoring between editors. The results have demonstrated a modest degree of inter-rater reliability.^9^ ^–^ ^12^

The inter-rater reliability of evaluations of performance by experts is confounded by idiosyncratic perceptions that are shaped by individual experiences, values, and priorities. Indeed, the preponderance of the literature argues that evaluation by experts is often subjective and nuanced.^2^ ^,^ ^4^ ^–^ ^6^ ^,^ ^8^ ^,^ ^10^ ^,^ ^13^ ^–^ ^17^ Cole et al proposed that the potential divergent perspectives among peer reviewers are often the result of “*real and legitimate differences of opinion among experts about what good science is,*”^14^ a concept supported by others.^15^ ^–^ ^17^ Capturing the nuanced and potentially divergent perspectives of reviewers allows editors to develop a holistic understanding of the value of a manuscript.^15^ This variability among editors’ perspectives limits the degree of reliability that can be achieved in assessing individual reviews.

The Special Issue’s editorial evaluation of reviews has traditionally depended upon a single, global five-point scale with anchors at the extremes (5 = high quality, 1 = low quality). A number of issues have been appreciated by the editors with this approach: 1) The website template only allowed for a single editor’s evaluation of a review; 2) scores of 1 and 2 were seldom used; and 3) no guidance was provided for editors to determine how to score on the five-point scale, resulting in a lack of valid evaluation data on which to base decisions pertaining to the quality of reviews.

Our objective in this initiative was to develop and study a scoring rubric for editors to assess the quality of a review with an emphasis on the degree to which it informs a holistic appreciation for the submission under consideration. Herein we describe the development, refinement, and pilot-testing of this rubric. Additionally, our reporting was grounded within the validity evidence framework suggested by Kane to inform the interpretations of scores generated by this tool.^18^

## METHODS

### Holistic Editor’s Scoring Rubric Development

This study involved graduate medical education (GME) submissions to the Special Issue and was determined to be exempt by the George Washington University Institutional Review Board.

There are several recurrent themes identified in the literature that appear important to developing an effective peer-review evaluation system. Such a system should be: 1) practical and simple to use^5^ ^,^ ^6^ ^,^ ^19^ ^,^ ^20^; 2) criterion referenced^4^ ^,^ ^5^ ^,^ ^20^; and 3) be able to capture differences in expert reviewers’ perspectives.^14^ ^,^ ^16^ ^,^ ^21^ ^–^ ^23^ To successfully operationalize an evaluation system, past works also suggest that rater training is necessary to ensure proper implementation.^3^ ^,^ ^10^ ^,^ ^13^ ^,^ ^20^ ^,^ ^24^ ^–^ ^28^

Prior to the production cycle for the 2020–2021 Special Issue the Council of Residency Directors in Emergency Medicine (CORD) guest editor and three associate editors discussed the need for an improved system for evaluating peer reviews. The use of a global five-point score has been shown to be practical in assessing reviews.^5^ ^,^ ^6^ By adding anchors to each point on the five-point scale based on quality as the criteria reference, Landkroon et al provided early validity evidence supporting its use.^6^ To define characteristics important to high-quality peer reviews in developing anchors for the current study’s five-point global scale, the editors reviewed the mission and vision statements of CORD,^29^ the literature relevant to peer-review scoring instruments and reviewer guidelines from four major medical journals.^30^ ^–^ ^33^ Through an iterative process, the editors defined qualities of an ideal review as one that provides the following: 1)–insights that reflect both the value to readership and alignment with the current literature; 2)–consideration of the appropriateness of the study method(s) and relevant tenets of education scholarship; and 3)–feedback that provides mentorship to authors on how to improve their manuscript as well as their own skill set.

**Figure 1.**
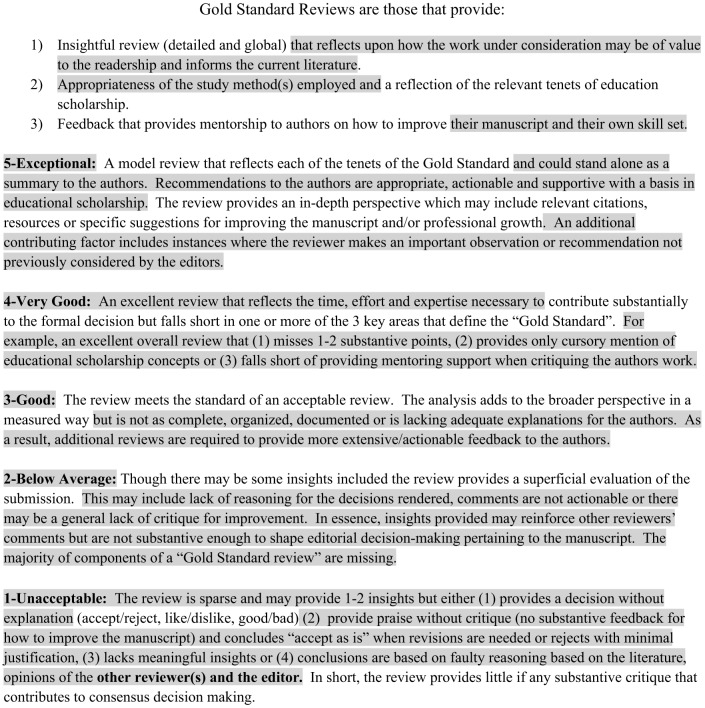
The holistic editor’s scoring rubric used for evaluation of peer review. The initial version of this rubric can be appreciated as the unshaded content. Subsequent additions made based on a pilot of the 14 initial reviews are denoted by the shaded areas (See “Preliminary Calibration Exercise.”) *CORD*, Council of Residency Directors in Emergency Medicine.

Through the same process, the editors determined that the anchors for the five-point scoring rubric should be based on these three provisions of a quality review as well as the degree to which the review informs the final evaluation of the submission under review (Figure 1). In other words, a review evaluation of five on this holistic editor’s scoring rubric (HESR) provides all three provisions of a quality review and could stand alone as the final evaluation of that submission (Gold standard review).

### Validity Assumptions

We used Kane’s framework to gather validity evidence for use of this instrument, which involved testing assumptions about scoring, generalization, extrapolation, and implication.^18^ Our first assumption involved the distribution of ratings or scores. In reviewing scores from the past few years with the traditional five-point scale, we observed limited use of the evaluation scale with skewing towards higher scores (>3); editors were hesitant to assign scores of 1 or 2 when appropriate. The wide variability of experience and expertise among the reviewers suggested that greater variability in scores should have been present. This skewed distribution could be attributed to a leniency bias, which is not uncommon in medical education evaluations.^23^ ^,^ ^34^ ^–^ ^37^ Our logic followed that for the HESR to be a valid reflection of peer-review performance, the peer-review evaluation scores must reflect the full range of peer-review performance. If successfully developed and implemented, the HESR peer-review evaluation scores would have a distribution where all rating options were used.

Our second assumption involved inferences about scoring, namely that the assigned HESR score for a peer review would be an accurate representation of the editor’s perspective of the quality and value of a peer review to editorial decision-making. If true at each score level, the associate and senior editors would be consistent with each other in applying the HESR for any given review.

Our third assumption had to do with implications that HESR scores would be used to inform the editorial decision-making process. In other words, highly rated reviews would have more value in decision-making, and as a result the reviewer’s decision recommendation (i.e. accept, revise, reject) would more closely align with the editor’s formal decision.

### Study Setting and Participants

The Special Issue was established in 2014 as an annual publication of *WestJEM* dedicated to educational research practice.^38^ Submissions related to GME were managed by a single guest editor and three associate editors. Peer reviewers for the Special Issue were recruited by the senior editor via the CORD and Clerkship Directors of Emergency Medicine listservs. The prerequisite for becoming a reviewer included recognition as an experienced educator and authorship of at least one scholarly educational study published in a peer-reviewed journal.

**Figure 2.**
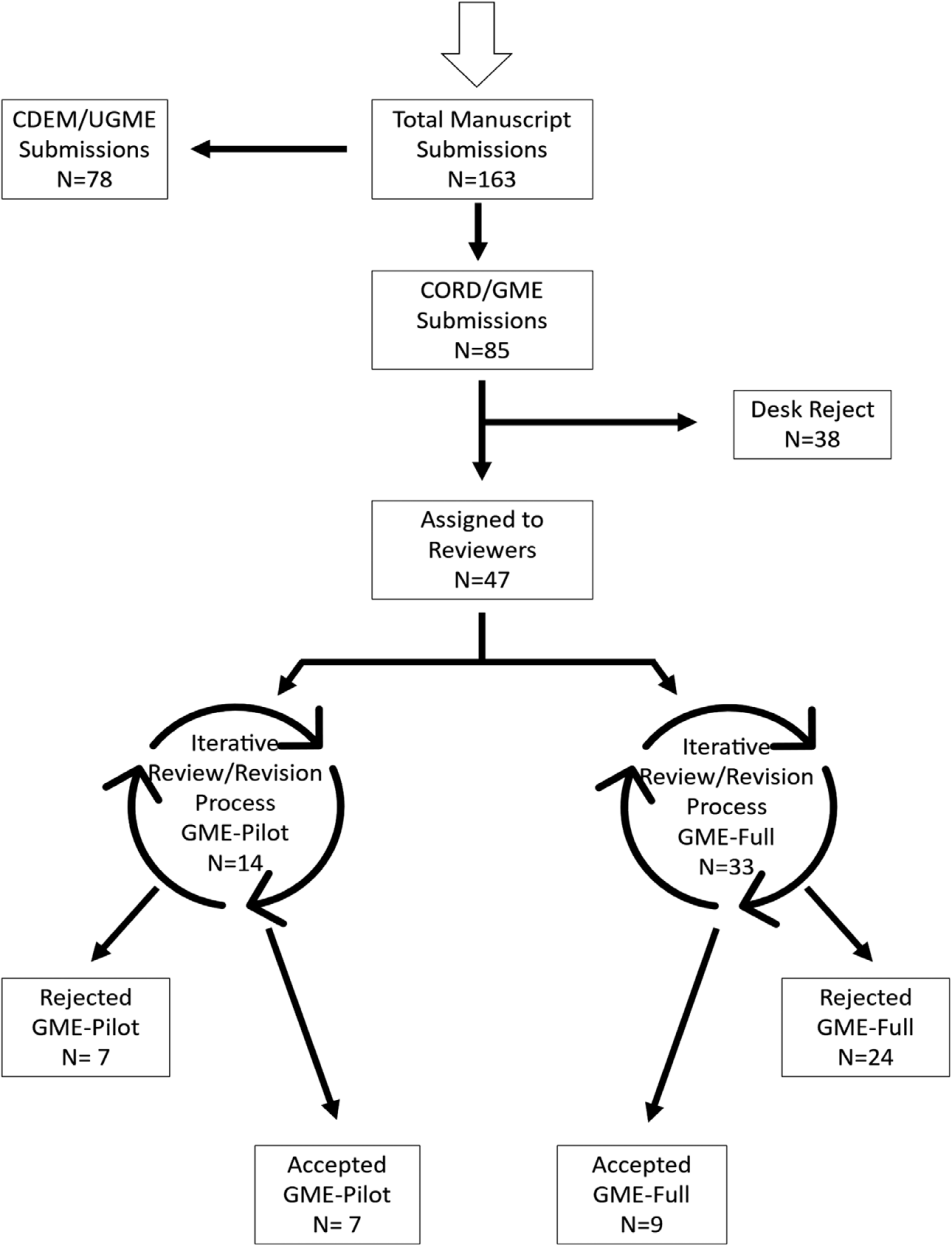
Flow chart showing the number of manuscripts submitted and processed during the 2020–21 submissions cycle for the *Western Journal of Emergency Medicine* Special Issue in Educational Research & Practice. *UGME*, undergraduate medical education; *CDEM*, Clerkship Directors of Emergency Medicine; *GME*, graduate medical education; *CORD*, Council of Residency Directors in Emergency Medicine.

Once a manuscript was submitted via the *WestJEM* submission portal, screening editors either approved the manuscript for peer review or chose to “desk-reject” the manuscript without review. Manuscripts that passed the screening process were then assigned to two external peer reviewers. In an iterative process, reviewers concluded their reviews with a recommendation to the associate editor who in turn made a recommendation to the senior editor for a formal decision. At each step in this process the choice was to reject, revise, or accept the manuscript. In those instances where revisions were requested and submitted, the final decision was either to accept or reject for publication (Figure 2). Reviewer assignment was random without regard to defined expertise (eg, statistics, specific methodology, topic under consideration).

Through the first five editions of the Special Issue, peer reviews were “rated” by associate and senior editors using the methods and instrumentation adopted from the parent *WestJEM* editorial board: a closed, internal evaluation system that used a global five-point semantic differential scale with labels at the extremes: 5 = high quality and 1 = low.

### Implementation of the Holistic Editor’s Scoring Rubric

The HESR was piloted during production of the 2020–2021 Special Issue. Manuscripts reviewed by the four CORD editors of the Special Issue were included in the pilot. To minimize issues of recall and maintain consistency, the editors all agreed to reflect upon the HESR just prior to scoring each peer review. Subsequently, each peer review received an independent score from an associate editor and the senior editor. Blinding was accomplished by having each editor report their review score to a third party. The third party (AM), who was not involved in the formal review process, maintained the secured database that linked the editor’s and associate editor’s HESR ratings for each review.

#### Preliminary Calibration Exercise (Pilot)

The editors paused to review their experience with the original HESR after the first 14 peer reviews of manuscripts had been scored. Comparisons between scores assigned by the senior and associate editors raised questions and concerns about the clarity of the scoring rubric, which warranted another round of revisions. Changes were made based on iterative discussions and consensus to improve the clarity of the rubric options and ratings. In addition, adjective descriptors that characterized each option were added as follows: 5-Excellent, 4-Very good, 3-Good, 2-Below average, and 1-Unacceptable (Figure 1). During the implementation stage, the final HESR was used to score the remaining 32 GME submissions during the 2020-21 Special Issue production cycle.

### Data Analysis

#### Assumption 1–Distribution of Evaluation Scores

Our first assumption was that a valid scoring mechanism of editorial evaluation of peer reviews should reflect the variability of quality and value of the reviews. The previous semantic differential rating system used by the Special Issue during a prior production cycle (2019–2020) did not reflect a high degree of variability in peer-review scores. In fact, the distribution of scores from this cycle appeared negatively skewed with scores clustered around “4” on the five-point semantic differential scale. Accordingly, one goal of the new HESR was for it to more accurately reflect the variability of the reviewer pool with regard to scholarly expertise through use of the entire evaluation scale. Using the “Explore” feature in IBM-SPSS version 28 (SPSS, Inc, Chicago, IL) to generate histograms, frequency distributions, and measures of variability, we compared three sets of peer-review scores: semantic differential ratings from the 2019–2020 Special Issue (baseline); the pilot CORD editor’s evaluation of peer reviews; and the full implementation of CORD editor’s evaluation of peer reviews using the revised HESR for the 2020–2021 edition.

#### Assumption 2–Inter-rater Reliability Between Evaluators of the Same Peer Reviews

Since the HESR provided clear criteria for five different levels of peer-review performance, we expected the HESR to generate reliable scores across editors. Accordingly, like Cicchetti, we compared inter-rater reliability among editors using the intraclass correlation coefficient (ICC).^39^ To complement our reliability evaluation, we also used three measures of agreement between associate and senior editors’ ratings of peer reviews for the CORD editorial team: percent of absolute and relative agreement, and the Spearman rho correlation for ordinal level data.^40^ The percentage of identical ratings is a measure of absolute agreement between raters, while the percentage of ratings in close proximity of each other (+1) is an indicator of within-rater consistency. The Spearman rho correlation provides an indicator of the strength of the relationship between the ratings across the two types of raters.^41^ We used the criteria from Schober et al. for interpreting the Spearman rho correlation (r of 0–0.10 = negligible; r of 0.10–0.39 = weak; r of 0.40–0.69 = moderate; r of 0.70–0.89 = strong, r of 0.90–1-very strong).^40^

The ICC model selected for this study is based on several assumptions. First, it is assumed that associate editors were randomly chosen from a larger pool and that the senior editor was fixed. Second, the design was not fully crossed, since not every review was rated by the same editors. Third, since one rating was the focus, rather than a series, the absolute agreement was thought to be the most appropriate ICC model. A final assumption was that since the ICC was being asked to represent the average of several coders, the “average measures” ICC was chosen. In summary, the ICC formula chosen for this study is a one-way random effects model reflecting absolute agreement and the unit of analysis related to average measures.^42^ We applied guidance from Cicchetti for interpreting the resulting ICC reliability indices (ICC of <.4 = poor reliability; ICC of .40–.59 = fair reliability; ICC of .60–.74 = good reliability; ICC of .75–1.0 = excellent reliability).^39^ Unfortunately, we were not able to perform comparable inter-rater reliability analyses for the prior Special Issue production cycle (2019–2020) due to the templated ability to provide only one editor’s score per manuscript.

#### Assumption 3–Implications or the Statistical Relationship Between Peer-Review Rating and the Editorial Decision

The collective editors’ evaluations of the peer review were assumed to be an indicator of its quality. If editors placed value on peer reviews due to their ability to inform the decision-making process, then higher quality peer reviews should have been more likely to agree with the editorial decisions than lower quality peer reviews. In this analysis, the categories of yes/no refer to whether the reviewer’s recommendation agreed with the formal manuscript decision. “Yes” designations were applied if the reviewer recommended the article be accepted, rejected or revised and the editorial decision made agreed with that recommendation. If the reviewer’s recommendation did not agree with the editor’s decision, this was categorized as a “No.” This is known as a parallel line of validity evidence according to Kane.^18^ We tested this hypothesis by averaging the senior and associate editors’ peer-review ratings and then categorizing these average ratings into five categories: (1–1.5); (2–2.5); (3–3.5); (4–4.5); and (5). Next, using a chi-square test of association we tested the relationship between the summary rating category and the reviewer’s agreement with the final decision (Did the reviewer’s recommendation agree with the final decision, yes or no?). If true, the authors posited that the higher the ratings by the editors on the quality of the peer review, the more likely their recommendations for the manuscript submission would agree with the actual formal decision. We applied the criteria from Hahs-Vaughn et al for interpreting the associated effect sizes from the chi square test of association (small effects = <0.10, medium effects = 0.30; and large effects are >0.50).^43^

We also evaluated the relationship between the HESR score and the reviewer’s agreement with the final decision using logistic regression analysis. For this analysis, we attempted to predict whether the reviewer recommendation would match the final editorial decision (yes or no) from the HESR scores assigned by each type of editor. Results of this test should provide a relative strength of the relationship between each type of editor’s rating and the editorial decision.

## RESULTS

The total number of manuscripts submitted for the 2021 Special Issue was 163. Of these, 85 were managed by the CORD editors. Thirty-eight submissions were desk-rejected by the editorial staff. Subsequently, 47 (55.3%) manuscripts were approved for peer review and 16 were published, for an acceptance rate of 18.8%. These 47 peer-reviewed manuscripts were the subject of this study, 14 during the pilot period and 33 during full implementation of the HESR (Figure 2).

Eighty-four peer reviewers reviewed an average of 1.84 manuscripts each (SD 1.34). About two-thirds of peer reviewers performed only one review (52/85; 61.2%), while an additional 34% (29/85) completed 2-4 reviews, and four individuals (4.8%) completed 5–7 reviews. The editors performed 91 evaluations of peer reviews, 27 at the pilot stage and 64 at the full implementation stage. The three associate editors performed 95 peer-review evaluations, 32 at the pilot stage and 63 at the full implementation stage (Table 1). There were 90 matched pairs of evaluations on the same peer review from both the senior and associate editor.

**Table 1.** Number and percentages of senior editor, associate editors, and reviewers involved in the production of the 2021 Special Issue by group. Included are the numbers of review evaluations performed and manuscripts processed by senior and associate editors and the numbers of peer reviewers and manuscripts they reviewed.

	CORD HESR Study						Total			
	Calibration-pilot			Implementation					
	Personnel	Review evals	Manuscripts	Personnel	Review evals	Manuscripts	Personnel	Review evals	Manuscripts
Senior editor	1	27	14	1	63	33	1	90	47
Associate editors	3	32	14	3	63	33	3	95	47
Reviewers*	32	N/A	14	69	N/A	33	84	N/A	47

* There were 84 total peer-reviewers who reviewed manuscripts during either the pilot or full implementation phase of this study. Fifteen of 32 reviewers participated only during the pilot while the other 17 contributed to reviewing at both stages of the project (pilot and full). Reviewers were not involved with using the Holistic Editor’s Scoring Rubric to assess their own peer-reviews.

*CORD*, Council of Residency Directors in Emergency Medicine; *HESR*, holistic editor’s scoring rubric; *evals*, evaluations.

### Distribution of Scores

**Figure 3.**
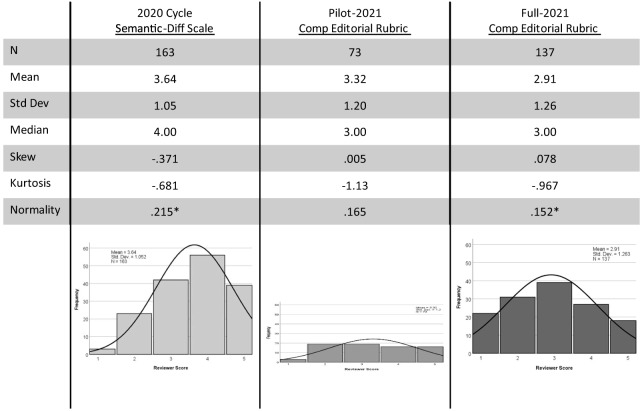
Comparison of the peer-review ratings distributions for two methods of editor evaluations across two Special Issue production cycles. The first method involved a 5-point semantic differential scale with labels only at the end points, which was used during the 2020 cycle. The second method involved the holistic editor’s scoring rubric used by the CORD editors at the pilot and full implementation stages of the 2021 production cycle. *CORD*, Council of Residency Directors in Emergency Medicine. *Data significantly deviate from normal distribution (*P* ≤ 0.001).

During the prior production cycle (2019–2020), 163 peer reviews were rated using a five-point semantic differential scale. The distribution of editors’ ratings of these reviews was shown to be negatively skewed (−0.371), which was caused by the underuse of the “1” rating and overuse of the “4” and “5” ratings (Figure 3). Contrasted with the semantic differential scale, the HESR distribution at both the pilot and full implementation stage had skewness closer to zero (0.005 and 0.078, respectively). The distribution during the pilot stage is considered a parallel distribution, since almost all response options were chosen equally (except for the “1” HESR rating). During the full implementation, negative skewness (0.078) almost disappeared as the distribution became more bell shaped, and kurtosis continued to suggest a distribution with symmetry (kurtosis = −0.967) (Figure 3).^44^

### Inter-rater Agreement

Ninety peer reviews were assessed with the HESR by both the senior editor and one of the three associate editors. The percentage of absolute agreement between the two types of editors’ ratings of peer reviews was 37.8% (Table 2). Nearly half (47.8%) of ratings were in disagreement by only one point. The associated Spearman rho correlation and r-squared for the ratings from the two types of assessors was 0.703 (R^2^ = 0.49). A Spearman rho of this magnitude is interpreted as bordering between a moderate and strong positive correlation or statistical relationship.^40^ Finally, the ICC between the associate and senior editors was 0.795. As interpreted by Cicchetti, an ICC of this magnitude is considered excellent in terms of clinical significance (between 0.75–1.00).^39^

**Table 2.** Results of logistic regression using the outcome, whether the reviewer recommendation matched with the final decision (yes or no), regressed on the predictors: associate and senior editor’s peer-review ratings.

Equation 1: Associate editor’s score as predictor for review rec/final dec match [93]									
							95% CI for EXP(B)		
	B	SE	Wald	df	Sig.	Exp(B)	Lower	Upper	% CC
Associate editor review score	.627	.184	11.571	1	<.001	1.871	1.304	2.685	67.7
Constant	−2.054	.631	10.587	1	.001	.128			
Equation 2: Senior editor score as predictor for reviewer rec/final dec match [N = 91]									
							95% CI for EXP(B)		
	B	SE	Wald	df	Sig.	Exp(B)	Lower	Upper	% CC
Senior editor review score	.935	.247	14.350	1	<.001	2.548	1.57	4.133	63.7
Constant	−2.664	.721	13.633	1	<.001	.070			
Equation 3: Associate & senior editor scores as predictor for reviewer rec/final dec match [N = 90]									
							95% CI for EXP(B)		
	B	SE	Wald	df	Sig.	Exp(B)	Lower	Upper	% CC
Associate editor review score	.137	.244	.314	1	.575	1.146	.711	1.849	66.7
Senior editor review score	.834	.313	7.084	1	.008	2.302	1.246	4.254	
Constant	−2.789	.765	13.300	1	<.001	.061			

### Implication of HESR Scores as Associated with and Predictors of Manuscript Outcomes

The chi-square test of association for the relationship between average peer-review ratings and the peer reviewer’s recommendation with the final manuscript decision was statistically significant (chi-square = 17.4, df = 4, *P* < 0.01, effect size = 0.44). The associated effect size of 0.44 is classified as a medium effect size according to Hahs-Vaughn et al who suggest that small effects are those ≤ 0.10, medium effects = 0.30, and large effects >0.50 (Table 3).^43^

**Table 3.** Reviewer summary rating grouped into 5 categories cross tabulated with whether the reviewer’s recommendation agreed with manuscript final decision (expected values are in parentheses) with chi square test of association* between these two variables.

		Did reviewer’s recommendation agree with final decision		
Reviewer summary rating*	N	Yes	No	Percent agreement
1.00	17	4 (8)	13 (9)	23.5
2.00	21	6 (10)	15 (11)	28.6
3.00	28	17 (14)	11 (14)	60.7
4.00	17	10 (8)	7 (9)	58.8
5.00	7	7 (3)	0 (4)	100
TOTAL	90	44 (44)	46 (46)	48.9
		*X* ^2^ = 17.4, df = 4, p = 0.006, es = 0.440		

* The minimum expected counts are 3.42. Cramer’s phi effect sizes are interpreted as ≤ 0.10 = small; 0.30 = medium; and ≥ 0.50 = large effects.

### Logistic Regression

For all logistic regression analyses, the tests for model coefficients were significant, suggesting that any one of the three formulas would improve our estimate of the probability that the peer-review recommendation matched the editorial decision. The Hosmer-Lemeshow tests were not significant, indicating that the models could be a good fit, and analyses of the scatter plots of predicted scores and residuals contributed to the conclusion that the analyses met the assumptions of normality and equal variance (statistics not shown).

Logistic regression analyses demonstrated that the associate editor’s HESR ratings were a significant predictor of the manuscript outcome: a successful match between the reviewer’s recommendation and the final decision for the manuscript. This was also true of the senior editor’s HESR ratings. However, because the ratings of the associate editor and senior editor were so highly correlated with each other (Spearman rho correlation = 0.703), once combined into one logistic regression model, only the senior editor’s ratings surfaced as a significant predictor.

Interpretation of the senior editor’s HESR ratings as a predictor suggests that the ratings contributed to improving the correct classification of predicted vs observed outcomes from 51.6% with no predictor to 63.7%. The adjusted odds ratio Exp(B) = 2.548 (95% confidence interval [CI] 1.570–4.133] can be interpreted as follows: “For every one step increase in the senior editor’s evaluation ratings, the risk of the outcome of a successful match between reviewer recommendation and the final decision increases by a factor of 2.548” (Table 2).

## DISCUSSION

Using Kane’s framework for validity evidence, this work tested three assumptions regarding the HESR as a novel means for editors to assess the quality of peer reviews of educational scholarship. The first assumption involved the distribution of editors’ ratings of reviews, finding that the HESR demonstrated greater symmetry in scores compared to a prior instrument used during the 2019 cycle. We conclude that leniency bias was likely limited by the criteria-referencing basis of this intervention, which may have resulted from clearer behavioral anchors of the instrument itself,^5^ ^,^ ^6^ ^,^ ^13^ improvements in rater training,^3^ ^,^ ^10^ ^,^ ^13^ ^,^ ^24^ ^–^ ^28^ and/or more intentional quality control among editors during the review process. This change in behavior may also reflect the practicality of the HESR since it was clearly being used in editorial evaluation of reviews.

The second assumption made infers that the assigned peer-review scores based on the HESR are an accurate representation of the editors’ perspective on quality and inform a holistic perspective on the submission. While the editors of the Special Issue had lower absolute agreement between the senior and associate editors (37.8%), they demonstrated excellent relative consistency reliability (ICC = 0.795) and correlations (Spearman rho = 0.703) between editors’ scores. In other words, their evaluations, while not identical, were internally consistent. This finding related to reliability supports the hypothesis that the HESR captures the editorial perception of quality as well as the degree to which peer review informs a holistic understanding of a submissions value.

Finally, the third assumptions made has to do with the implication that scores are used to inform the editorial decision-making process. This is substantiated by the finding that the ratings on the HESR corresponded to the reviewer’s agreement with the manuscript’s final disposition. The higher the peer-review evaluation of quality by HESR scoring, the higher the correlation between the reviewer disposition recommendation and the manuscript’s formal outcome. Although this is to be expected, the fact that it holds true in this instance demonstrates that editors value and rate reviews higher when they contribute substantially to the editorial decision.^18^

## LIMITATIONS AND FUTURE STUDIES

These findings should be interpreted in the context of several limitations. Traditionally, the Special Issue has not blinded its senior and associate editors to the identity of the reviewers. This raises the potential for bias if editors recognized peer reviewers’ names, which could conceivably have impacted the ratings of more familiar peer reviewers. Second, the editors in this study had regular discussions regarding the use and interpretation of this scoring instrument. Given the centrality of rater training in the use of any evaluation instrument, future work will help to determine whether the performance of the HESR and lack of skewness in scoring persists beyond the editorial focus associated with this study.

Most importantly, this study is based on a single cycle of an annual specialty-related publication focused on health professions-education topics with a small number of editors and reviews. Future studies should focus on assessing additional validity evidence supporting the HESR’s use as well as varying journal environments with larger numbers of editors and reviews. Our results are most likely to generalize to specialty-specific education journals whose approach is similar to that presented in this study. Our findings are less likely to generalize to journals that take an alternative approach such as those that bring together a diverse set of reviewers based on expertise (eg, methodology, psychometrics, content, etc) to assess specific components of the submission.

## CONCLUSION

A holistic understanding of the value of a scholarly submission requires an iterative process informed by the expert perspective of reviewers that is often subjective and nuanced. The holistic editor’s scoring rubric was developed as a practical approach to editorial evaluation of the quality of a review and the degree to which it informs the formal editorial decision. By studying a priori assumptions related to the development and use of the HESR. including distributions of evaluation scores, inter-rater reliability between evaluators of the same peer reviews and the statistical relationship between peer-review rating and the editorial decision, this study provides validity evidence supporting the use of the HESR. Future work should focus on further defining the value and limitations of the HESR.
